# Active Region Overheating in Pulsed Quantum Cascade Lasers: Effects of Nonequilibrium Heat Dissipation on Laser Performance

**DOI:** 10.3390/nano13232994

**Published:** 2023-11-22

**Authors:** Ivan I. Vrubel, Evgeniia D. Cherotchenko, Dmitry A. Mikhailov, Dmitrii V. Chistyakov, Aleksandr V. Abramov, Vladislav V. Dudelev, Grigorii S. Sokolovskii

**Affiliations:** Ioffe Institute, Politekhnicheskaya St. 26, 194021 St. Petersburg, Russia

**Keywords:** quantum cascade laser, joule heating, CW-mode, heat equation

## Abstract

Mid IR Quantum cascade lasers are of high interest for the scientific community due to their unique applications. However, the QCL designs require careful engineering to overcome some crucial disadvantages. One of them is active region (ARn) overheating, which significantly affects laser characteristics, even in the pulsed mode. In this work, we consider the effects related to the nonequilibrium temperature distribution when thermal resistance formalism is irrelevant. We employ the heat equation and discuss the possible limitations and structural features stemming from the chemical composition of the ARn. We show that the presence of solid solutions in the ARn structure fundamentally limits the heat dissipation in pulsed and CW regimes due to their low thermal conductivity compared with binary compounds. Also, the QCL postgrowths affect the thermal properties of a device closer to CW mode, while it is by far less important in the short-pulsed mode.

## 1. Introduction

The idea of quantum cascade lasers appeared as early as 1971 [1], and now they are the most promising platform for mid-IR sources and their applications. The latter vary from gas-sensing, industrial process control and environmental monitoring technologies to telecommunication and infrared countermeasures. Some of these applications utilize high-power laser emission, which in fact appears to be a tricky challenge. As discussed in [2,3], QCLs are less efficient compared with near-IR laser diodes, and therefore, only a small part of the input energy is converted into light. The major part is released via heating, which in turn degrades laser performance. Currently, wall-plug efficiencies (WPE) for mid-IR QCL at room temperatures in pulsed and CW modes reach 31% and 22%, respectively [4,5]. The reason for such unimpressive results lies in the internal heating dynamics of a laser. The thermal conductivity of a QCL ARn is essentially anisotropic and very low in the growth direction. The lower thermal conductivity is due to the higher temperature of ARn achieved during CW operation [6], which leads to emission degradation and lower WPE. Obviously, the same trend is valid for a pulsed regime.

The improvement of power and efficiency in laser performance involves thorough material engineering [7,8], and, even more importantly, careful thermal analysis and corresponding structural engineering. The latter includes the study of QCL geometry and ARn thermal conductivity [9], the effects of cladding and heat sink form factors affecting the thermal resistance [10], and the design of external cooling and its efficiency [11]. In turn, the experimental thermal analysis and management techniques are quite complicated and mostly do not allow to study processes inside the AR. General methods, like CCD thermoreflectance [12,13,14], scan only the facet, which is not informative about the inner processes. A few experiments allow the determination of ARn electrical properties [15], from which one can indirectly draw some conclusions about thermal properties. In this work, we discuss the fundamental limitations on the QCL performance arising from temperature modeling and show that the thermal properties in the short-pulsed regime are insensitive to the laser design, while the main features of the laser engineering play a major role only when closer to CW mode.

## 2. Results

### 2.1. Experiment

**Figure 1:**
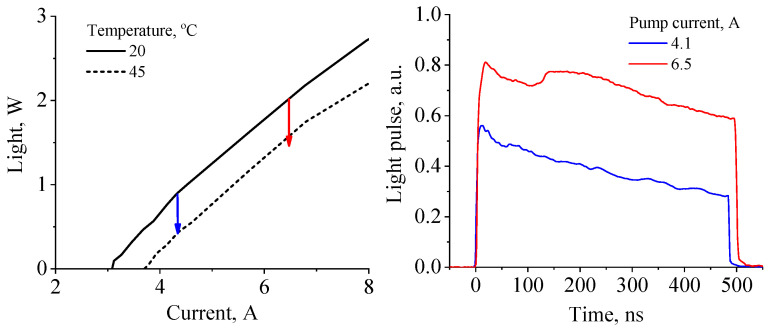
Upper panel: typical QCL light–current characteristics measured at two heat sink temperatures in a pulsed regime. Lower panel: time traces of light output of a QCL pumped by current pulses of two specified magnitudes. The time-dependent degradation of the light intensity related to the heating is also depicted in the upper panel using vertical arrows of corresponding color.

In this work, we used QCL samples similar to the ones used in ref. [7]. The active region comprised a superlattice of alternating **In${}_{0.53}$Ga${}_{0.47}$As**/Al${}_{0.48}$In${}_{0.52}$As layers lattice-matched to the InP substrate [16]. The thicknesses of these layers were **2.4**/2.4/**2.6**/2.1 /**2.6**/1.8/**2.7**/1.6/**2.9**/1.7/**3.1**/2.5/**4.4**/ 1.2/**5.2**/1.2/**5.3**/1.0/**1.7**/4.3 nm. The structure was grown on an InP substrate with a doping level of $1-3\times 10^{17}$ cm${}^{-3}$. A laser waveguide core consisted sequentially of a 0.5 μm thick In${}_{0.53}$Ga${}_{0.47}$As, 50 quantum cascades, and a thin In${}_{0.53}$Ga${}_{0.47}$As 50 nm thick layer. An InP waveguide plate with a thickness of 4 μm and a doping level of $1\times 10^{17}$ cm${}^{-3}$ was grown on top of the core. The epitaxial growth ended with a 20 nm thick contact layer. A detailed description of the technological process is given in ref. [7]. The heterostructure was processed in QCL chips with 40 μm stripes and 3 mm cavity lengths. All samples were tested under 500 ns pulsed pumping with a 12 kHz repetition rate. Here, we consider different QCL samples operating under varying pump current pulses at two given temperatures: 20 ${}^{\circ }$C and 45 ${}^{\circ }$C for the heat sink. In the very beginning of the pump pulse, the temperature of the ARn is close to the temperature of the thermal reservoir. Joule heat continuously releasing under operation results in an increase in phonon-assisted relaxation rates, which in turn lead to degradation of the laser output intensity with time. In Figure 1, we show the typical light–current characteristics (upper panel) and time-dependent pulse intensities (lower panel) measured in this experiment. Red and blue curves at the lower panel show the pulse evolution at two different current amplitudes. Red and blue arrows at the upper panel relate the maximum and minimum light intensity for both values of the pump magnitude. We chose 20 ${}^{\circ }$C and 45 ${}^{\circ }$C degrees for the ARn temperature initial conditions based on the estimations in adiabatic approximation, when there is no heat transfer between the ARn and claddings or any other parts surrounding the structure. Indeed, it appears that the degradation of the light intensity at 20 ${}^{\circ }$C stops in the vicinity of the light–current characteristic measured at a temperature of 45 ${}^{\circ }$C. Thus, it is reasonable to deduce that the overheating of a QCL ARn is as high as approximately 25 K at the end of a 500 ns pulse. All three types of structures from ref. [7] were used in this experiment, and they exhibit almost the same slope of light pulse degradation (see Appendix B).

### 2.2. Modeling

To reproduce the thermal behavior of the QCL structure, we employ the time-dependent two-dimensional heat equation.

(1) $$c\rho \frac{\partial T(x,z,t)}{\partial t}-\kappa \left(\frac{\partial^{2}}{\partial x^{2}}+\frac{\partial^{2}}{\partial z^{2}}\right)T\left(x,z,t\right)=q_{v}\left(x,z,t\right)$$

where c(x,z), ρ(x,z) and κ(x,z) are spatial functions, characterizing the specific heat capacity, density and thermal conductivity of a QCL design, and T(x,z,t) and $q_{v}\left(x,z,t\right)$ are time-dependent two-dimensional functions of temperature distribution and volumetric heat sources. A detailed analysis of quantum efficiency and QCL structural properties [7,17,18] allows us to deduce that at least 80% of heat generation occurs inside the ARn. Thus, we approximate the value of volumetric power source as

(2) $$q_{v}=\frac{U_{b}I_{pump}}{V_{ARn}},$$

where *V*${}_{ARn}$ is an active region volume, $I_{pump}$ is a pump current, and $U_{b}$ is a bias voltage. The measured V(I) curves are presented in Figure 2 of ref. [7]. In the considered range of currents (3–6 A), the voltage magnitude varies from 9 to 12 volts for different samples, so in the current manuscript, we use $U_{b}$ = 10 V without loss of generality in estimations. Finally, one can easily obtain $q_{v}\approx 10^{14}$ W/m${}^{3}$, which is a typical value for QCL [19].

**Figure 2:**
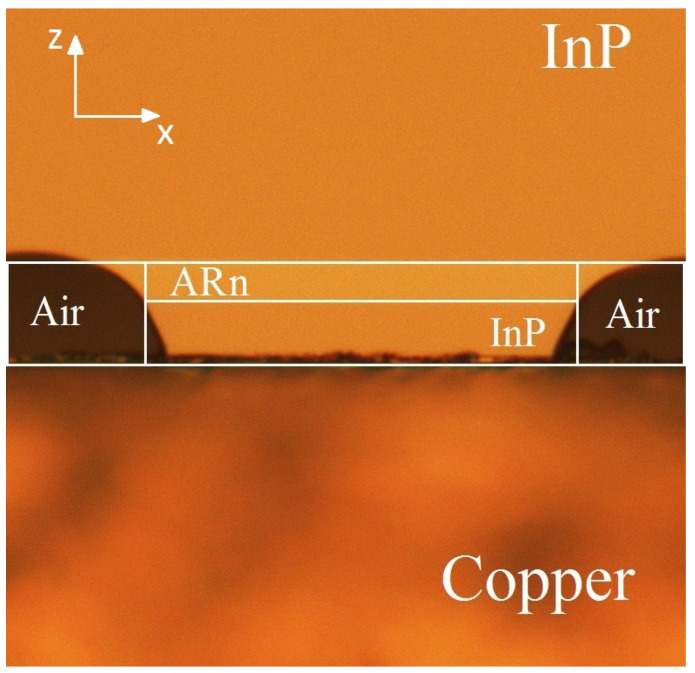
The image of a QCL structure taken with an optical microscope with a schematic of simplifications made for numerical modeling. In the latter, the ARn is considered as uniform media with averaged thermal parameters. For this calculation, ARn properties are taken similar to InAs, except for thermal conductivity.

The spatial model providing a relevant description of heat transfer processes is depicted in Figure 2. We use a two-dimensional map with domains mimicking thermal properties of InP claddings, copper heat sink, and ARn correspondingly (see Table 1). Here, we consider the direct modeling of the ARn heterostructure to be unnecessary for our goals, so for simplicity, the ARn heat capacity and density are set equal to that of InAs. The latter parameters vary by +/− 10% for any III-V compound [20]; thus, this approximation appears to be valid. The only unknown parameter of the modeled system is the ARn thermal conductivity, which significantly decrease in solid solution comprising fluctuating component inclusion [21,22] and plenty of interfaces [23,24]. So, we simply adjust it to the experimental data.

The values of thermal conductivities presented in Table 1 are, in principle, thermally dependent, especially for semiconducting InP and the active region. This appears to be extremely important when modeling QCL operation in the broad thermal range, e.g., from cryogenic to room temperatures [19]. However, in the temperature domain relevant to the performed experiment (293–318 K), the variation in thermal conductivity is about 10% [19,25] and can be neglected during modeling.

**Figure 3:**
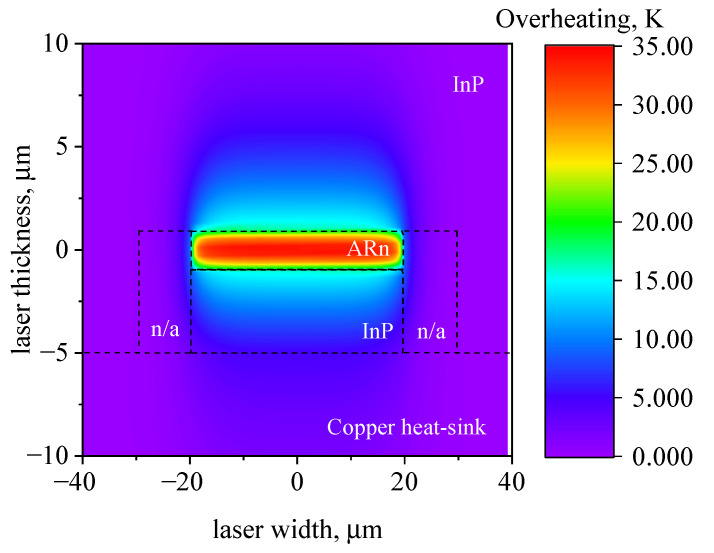
Heatmap of QCL cross section after 500 ns 6.5 A pump pulse. “n/a” indicates the regions with air parameters. The numerical calculation was performed with the real structure dimensions, indicated in the main text and ref. [7].

As mentioned above, the expected ARn overheating value is about 25 K. In order to obtain such a value during modeling, the effective ARn thermal conductivity was calibrated and set to 0.07 W (cm·K)${}^{-1}$, which is 10 % of that for InP [25]. The heat distribution map after 500 ns of 6.5 A pump pulse is depicted in Figure 3. The color indicates the heatmap in the structure fitted to the data in Figure 2. The zero of the coordinates is in the middle of the two-dimensional ARn. It can be clearly seen that the energy released in a QCL during 500 ns operation is confined in the vicinity of an ARn. The part of heat dissipated out from an ARn is approximately a half of the released amount. Also, at the end of the 500 ns pulse, the typical thermal diffusion length (the characteristic thermal spread length) was only 5 μm; hence, we conclude that the system is far from the steady-state regime when the thermal resistance formalism is valid. To achieve a pure steady-state regime, the ARn should heat up the area to as large as about 50 μm, with a diffusivity constant of InP.

**Figure 4:**
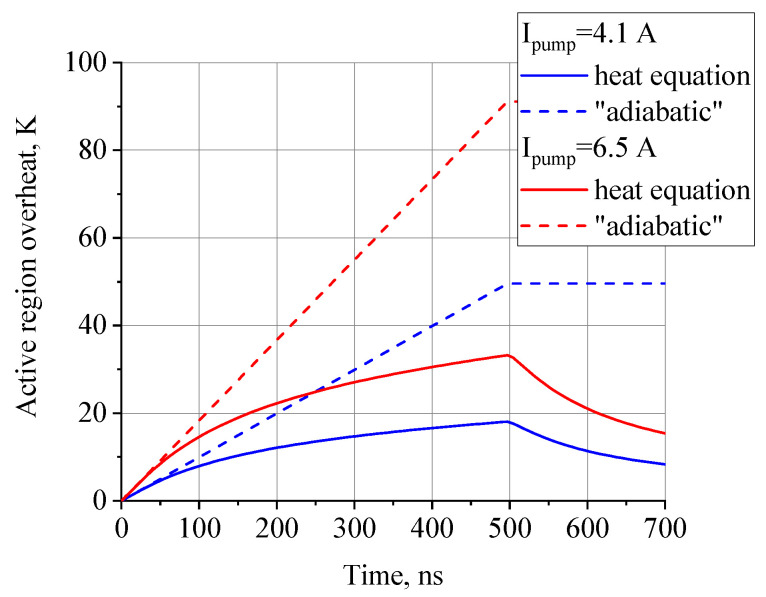
Heating dynamics of an ARn center under operation with two specific values of pump current. The pump pulse length is 500 ns. The dashed lines represent the adiabatic estimations, without dissipation from ARn. One can see that during the first 50 ns, QCL heating dynamics shows very little difference from the adiabatic regime, proving that under short-pulsed conditions there is quite low thermal dissipation.

The temperature profile of the ARn center is depicted in Figure 4. One can find that in the very beginning of a pulse (below 50–100 ns), the energy release can be considered as adiabatic. So, here, we can estimate that at the end of the modeled 6.5 A current pulse, the real temperature is thrice as high as the one in adiabatic approximation.

## 3. Discussion

### 3.1. ARn Thermal Conductivity Evaluation

As it follows from our modeling results, the effective thermal conductivity of InGaAs/AlInAs ARn in the growth direction is about 0.07 W(cm·K)${}^{-1}$, which is 5–10 times [23] lower than that for III-V pure bulk materials, such as GaAs (0.55 W(cm·K)${}^{-1}$), InAs (0.27 W(cm·K)${}^{-1}$), InP (0.68 W(cm·K)${}^{-1}$). The main reasons for such a drastic drop are the decrease in heat transfer due to the interface properties and the thermal properties of the solid solution. The rigorous consideration of interface properties, for example, with acoustic mismatch (AMM) and diffuse mismatch models (DMM), allows one to conclude that when a layer thickness is larger than a phonon mean free path, the interlayer heat transfer does not change significantly [22,23,24,26]. The main factor limiting in-plane thermal conductivity is a quality of interfaces (most likely roughness). Obviously, the flat interface induces more effective heat conductivity inside the layer compared with the rough one, allowing backscattering of the phonon. Also, a layer with diffusive (rough) interface lowers the transfer when the layer thickness becomes smaller. However, this effect also does not make much contribution to the heat transfer suppression. As a result, any addition of interfaces characterized by different fabrication technologies [22] reduces this parameter by a cofactor of 1.05 to 2.5. So, the Kapitza resistance between ARn layers has an upper limit of 0.1 m${}^{2}$K GW${}^{-1}$ and is negligible compared with the intrinsic solid solution resistances [23], even for 2 nm thick layers.

Thus, we consider the solid solution properties as the main factor of the heat transfer drop. The experimental research unambiguously demonstrated [22] that the thermal conductivity of solid solutions In${}_{0.5}$Ga${}_{0.5}$As and In${}_{0.5}$Al${}_{0.5}$As is about 0.02–0.04 W(cm·K)${}^{-1}$. Earlier studies [21,27] support the idea that the thermal conductivity of III-V solid solutions mostly decreases due to isovalent substitutions in the lattice.

### 3.2. Fundamental Limit on the Efficiency of Heat Transfer from ARn: Analytical Evaluation

As it was highlighted in the previous sections, an ARn thermal conductivity is characterized by the lowest value compared with other (pure) materials used in QCL structure. This means that an ARn itself is a bottleneck for the effective thermal management of QCL. As it follows from our own data and literature review, the main contribution to the thermal conductivity degradation comes from the use of solid solutions of III-V materials. The secondary effects are due to interfaces in the structure of ARn and heat sink configuration.

Now, we consider the physical aspects of the main challenge of QCL thermal management in a simple semianalytical manner. The pure binary bulk III-V materials manifest the value of thermal conductivity on the order of 0.5 W(cm·K)${}^{-1}$ at room temperature. On the one hand, this value is temperature-dependent, and on the other hand, it is rather stable with respect to the trace amount of impurities in the raw materials [21]. This allows us to conclude that in a pure binary III-V compound, the main effect limiting heat dissipation is a phonon–phonon scattering.

Assume that the macroscopic heat transfer is an envelop function characterized by microscopic process of phonon diffusion, with the thermal diffusion length given by Einstein relation:

(3) $$L_{diff}=\sqrt{2\alpha t}$$

where $\alpha =\frac{\kappa }{\rho c}$ [cm${}^{2}$·s${}^{-1}$] is a thermal diffusivity. At the same time, the diffusion length of an individual phonon with time *t* (s) assuming purely random walk can be assessed via the following formula:

(4) $$L_{diff}=\sqrt{\lambda \nu t}$$

where λ is a mean free path of the phonon (cm), ν is the speed of sound (cm·s${}^{-1}$). Based on the initial assumption, we equate thermal diffusion lengths derived via macro- and microscopic approaches and obtain the intrinsic (natural) mean free path for effective phonon mode. In pure InAs and GaAs, $\lambda_{nat}$ is estimated to be ∼10 nm.

If a pure semiconductor is diluted with impurity ions in the solid solution, the contaminating ions reduce the mean free path according to the following assessment:

(5) $$\frac{1}{\lambda_{tot}}=\frac{1}{\lambda_{nat}}+\frac{1}{\lambda_{imp}}=\frac{1}{\lambda_{nat}}+\sigma_{imp}N_{imp}=\frac{1}{\lambda_{nat}}+\sigma_{imp}N_{0}x$$

where $\sigma_{imp}$ (cm${}^{2}$) is a cross section of phonon-impurity scattering, $N_{imp}$ (cm${}^{-3}$) is a concentration of unit cells accommodating impurity ions, *x* is a ratio in the chemical compound formula (e.g., In${}_{1-x}$Ga${}_{x}$As) and $N_{0}$ (cm${}^{-3}$) is a concentration of unit cells in the initial lattice (4.4 × 10${}^{21}$ cm${}^{-3}$ for InAs).

Rewriting Equations (3) and (4), one can easily obtain the following expression for the thermal conductivity:

(6) $$\kappa =\frac{c\rho \nu }{2}\frac{\lambda_{nat}}{1+\sigma_{imp}N_{0}x\lambda_{nat}}$$

Thus, the degradation rate of the binary compound thermal conductivity with contamination in the low-impurity concentration limit reads:

(7) $$\frac{d\kappa }{dx}=\frac{c\rho \nu }{2}\frac{-\sigma_{imp}N_{0}\lambda_{nat}^{2}}{{\left(1+\sigma_{imp}N_{0}x\lambda_{nat}\right)}^{2}}$$

**Figure 5:**
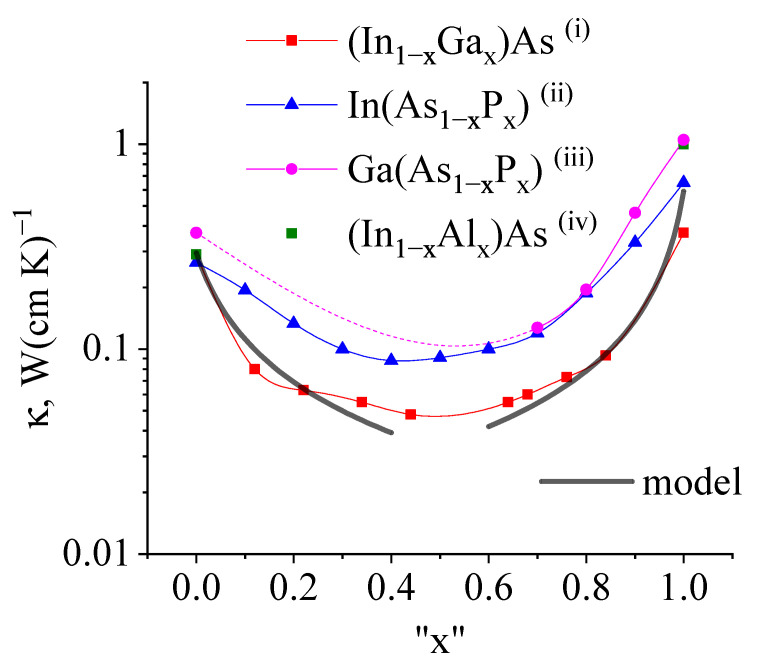
The log-scale dependence of the thermal conductivity on the III-V compounds’ chemical formula adopted from ref. [29]–(i), ref. [21]–(ii), ref. [30]–(iii), initially collected by Goryunova [27], and ref. [28]–(iv). “x" is the dimensionless impurity mole fraction. One can easily see that the binary compound shows better thermal characteristics than any solid solution. The dashed magenta line indicates the numerical interpolation due to the lack of experimental data for Ga(As${}_{1-x}$P${}_{x}$). The solid black line indicates the log-scale thermal conductivity degradation of In${}_{1-x}$Ga${}_{x}$, as calculated according to Equation (6). The theoretical estimations are quite close to the experimental results.

The only unknown value to guess in the Equations (6) and (7) is the cross section characterizing phonon-impurity scattering $\sigma_{imp}$, which we equal to the square of the lattice constant ($\sigma_{imp}$ = 3.6 × 10${}^{-15}$ cm${}^{2}$ for InAs). The thermal conductivity degradation profile and its slope in the limit of the low-impurity concentration for the guessed cross-section value are depicted in Figure 5. The plotted profile is typical for III-V compounds (see, e.g., refs. [21,27,28]). The comparison of the theoretically estimated slope in the low-concentration limit and experimentally assessed values collected in ref. [27] are presented in Table 2, showing quantitative and qualitative agreement. One can find that different III-V solid solutions show a similar behavior of thermal conductivity. The theoretical estimation for κ(x=0.5) in the Table 2 is performed with direct substitution of *x* = 0.5 to the Equation (6), which turns out to be formally inapplicable, as the theory assumes only a small fraction of impurity to the binary compound. This results in a significant deviation from what one obtains in the experiment. Regardless, Equations (6) and (7) are still valid for rough quantitative estimations.

### 3.3. CW Regime and Structure Safety

**Figure 6:**
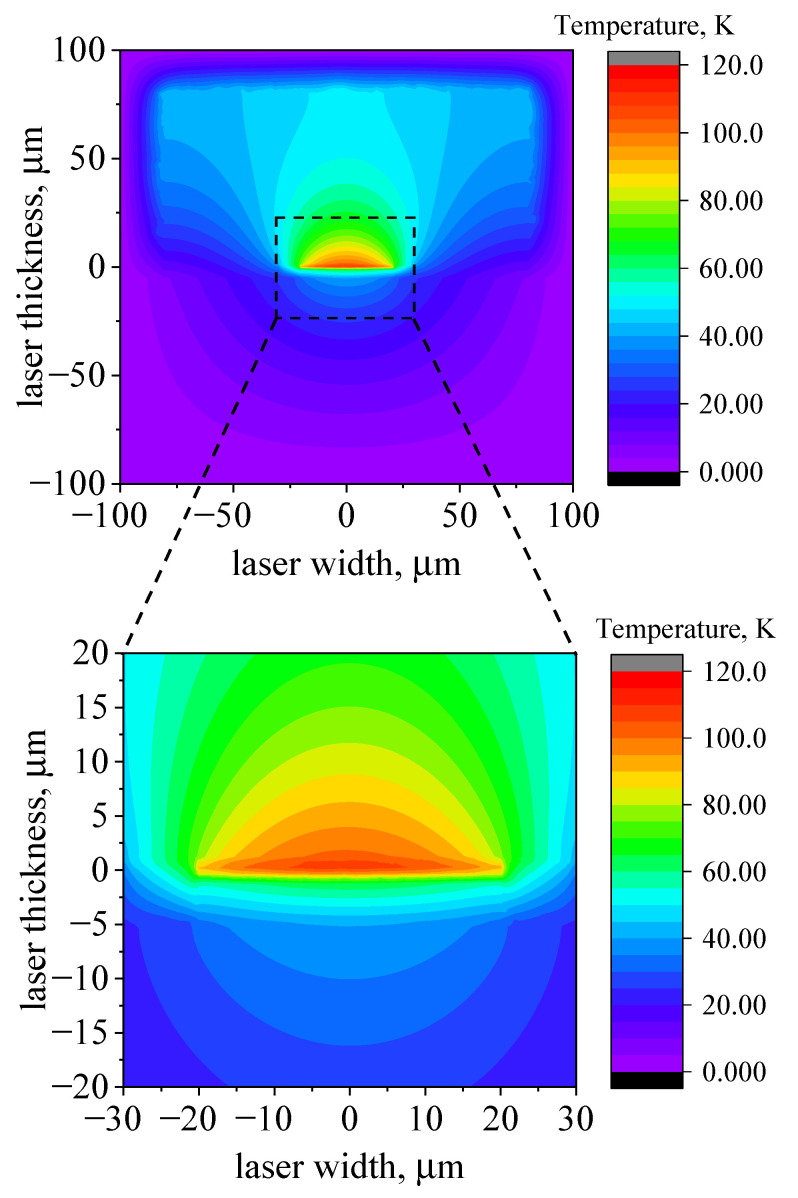
Heat map of QCL device schematically similar to the one in Figure 2 under CW pump current of 6.5 A. The copper heat sink starts at the −5 μm level. The size of a sample for this calculation is 0.2 × 0.2 mm. The upper panel shows the reached steady-state operational mode, with the isotherms of heat spreading. The lower panel is a zoomed ARn from the upper panel, shown by the black rectangle.

Taking into account the discussed results, we continue our modeling and consider the ability to reach the continuous-wave regime for the mentioned structure. Here, we neglect the technical details of heat transfer to the coolant, assuming that there is a reservoir supporting room temperature on the boundaries of the modeled box with the given size. Adopting these simplifications, we run the simulation for a long enough period and control if the system reaches steady state. The results are given in Figure 6. Calculations show that after about 10 μs of steady-state pumping, the temperature of a central ARn part stabilizes, with overheating of about 100 K (see lower panel of Figure 6).

These results can be generalized by introducing an equivalent thermal resistance scheme comprising comprehensible primitives. To proceed, we make the following assumptions:

- The device operates at room temperature (RT);
- The temperature on the outer surface of the heat sink is stabilized equal to RT;
- The distribution of the temperature inside an ARn is neglected;
- The heat leakage from the sides of the laser ridge is neglected;
- The geometry of an ARn is needle-like, meaning that its linear dimensions in the XZ plane(facet plane) are negligible compared with the copper dimensions (see Figure 2). This allows us to consider the cylindrical distribution of isotherms in the copper heat sink. The latter agrees well with Figure 6.

**Figure A1:**
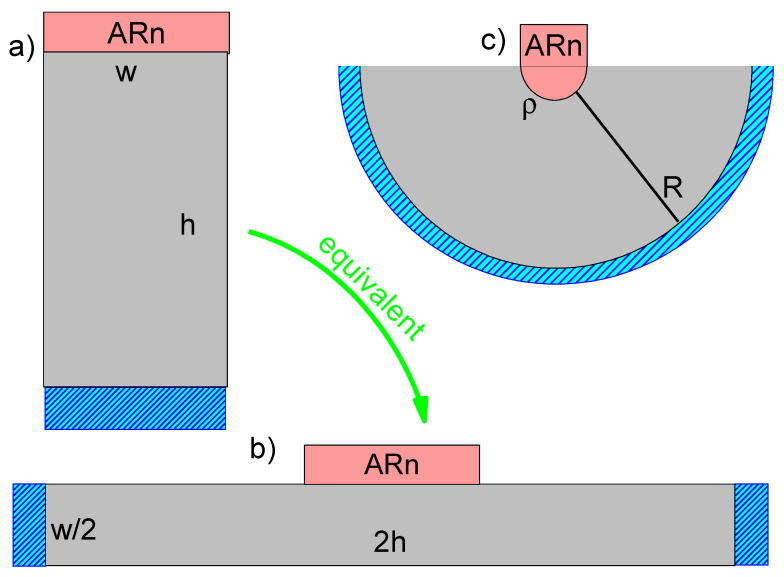
Schema of flat (**a**,**b**) and cylindrical (**c**) heat spreaders: red bars denote AR, blue bars denote the contact of the heat spreader with temperature “reservour” and gray bars depict heat spreader medium.

Under these assumptions, the corresponding serially connected primitives are presented in Figure A1.

In a real device, the ARn may be separated from the coolant by the two thermal resistors, the InP rod and copper cylinder [31], which are coupled in the series circuit. The thermal resistance of the first part is

(8) $$T_{InP}\approx T_{1D}=\frac{P}{\kappa_{InP}Lw}h$$

The second component of heat spreader reads as

(9) $$T_{Cu}\approx T_{rad}=2\frac{P}{\kappa_{Cu}\pi L}ln\left(\frac{R}{\rho }\right)$$

where h,w,R and ρ are linear and radial dimensions indicated in Figure A1a–c, κ is thermal conductivity and *L* is QCL length. Substituting realistic values for the latter ones, we obtain $R_{InP}\approx 0.5\left[\frac{K}{W}\right]P$ and $R_{Cu}\approx 2\left[\frac{K}{W}\right]P$ for *w* = 40 μm, *L* = 3 mm, *h* = 4 μm, $\kappa_{InP}$ = 0.68 W(cm·K)${}^{-1}$, $\kappa_{Cu}$ = 4.0 W(cm·K)${}^{-1}$, *R* = 1 mm, ρ = 40 μm. The details for these expressions can be found in Appendix A.

It can be seen in Figure 6 that the overheating of the upper and lower sides of the 4 μm InP rod is about 70 K and 50 K, respectively, which results in an almost linear 20 K temperature gradient. The remaining overheating of 50 K is distributed over the copper heat sink. The ratio of these two thermal differences (20 K/50 K) is close to the ratio of assessed thermal resistances, 0.5 $\frac{K}{W}$/2 $\frac{K}{W}$.

In the estimations above, we did not consider the ARn thermal properties. Apparently, its thermal conductivity is an order of magnitude lower than that value for pure InP. Taking into account the 2 μm thickness of an ARn, this creates an additional temperature increase in the center of QCL and significant nonuniformity in the temperature distribution over QCL volume. Such drastic localized thermal variations create strong lattice strains; therefore, it should be a parameter for monitoring in experiments to prevent potential device damage [31,32].

## 4. Conclusions

In this work, we discuss the fundamental limitations on the QCL performance related to Joule heating. We measured the intensity degradation in InGaAs/InAlAs QCL in a short-pulse regime and found that it follows a trend typical for adiabatic heat release at the very beginning of the pump pulse. The heat equation modeling reveals that the leakage of Joule heat released in ARn starts approximately 50–100 ns after the pump pulse begins, which is related to the creation of the temperature gradient in the vicinity of ARn. The process of heating accumulation takes tens of microseconds and finishes when the steady-state thermal distribution is reached. The latter corresponds to a QCL CW mode and allows rather simple description by thermal resistance approximation.

In both pulsed and CW regimes, the cornerstone of the effective thermal management is the thermal conductivity of an ARn, which suffers from the QCL structure interfaces and utilization of III-V solid solutions with compositions far from the binary compounds. In particular, in our calculations, we assessed the effective value of 0.07 W (cm·K)${}^{-1}$. The low effective thermal conductivity of an ARn in turn leads to even higher nonuniformity in temperature distribution, which adds more strains to the device construction.

## Figures and Tables

**Table 1:** Material parameters used for QCL overheating modeling.

	Heat Capacity, J (g K)${}^{-1}$	Density, g cm${}^{-3}$	Thermal Conductivity, W (cm·K)${}^{-1}$
InP	0.31	4.8	0.68
Cu	0.40	8.9	4.00
ARn	0.31	5.5	0.07

**Table 2:** The experimentally assessed parameters illustrated in Figure 5 for some III-V solid solutions: thermal conductivity degradation rate for almost pure material (x→0) (second column) and the relative deterioration (third column) of the thermal conductivity for a half-diluted solution and pure binary compound. The experimental data are taken from ref. [27]. The theoretical estimation for κ(x=0.5) is performed with direct substitution of *x* = 0.5 to Equation (6).

Solid Solution	dκ/dx (x→0), W(cm·K)${}^{-1}$	$\frac{\kappa (x=0.5)}{\kappa (x=0.0)}$, %
In${}_{1-x}$Ga${}_{x}$As	2	18
In${}_{x}$Ga${}_{1-x}$As	2	22
GaAs${}_{1-x}$P${}_{x}$	5	11
InAs${}_{1-x}$P${}_{x}$	1	30
InAs${}_{x}$P${}_{1-x}$	4	14
Theory (In${}_{1-x}$Ga${}_{x}$As)	4.0 (Equation (7))	11 (Equation (6))

**Figure A2:**
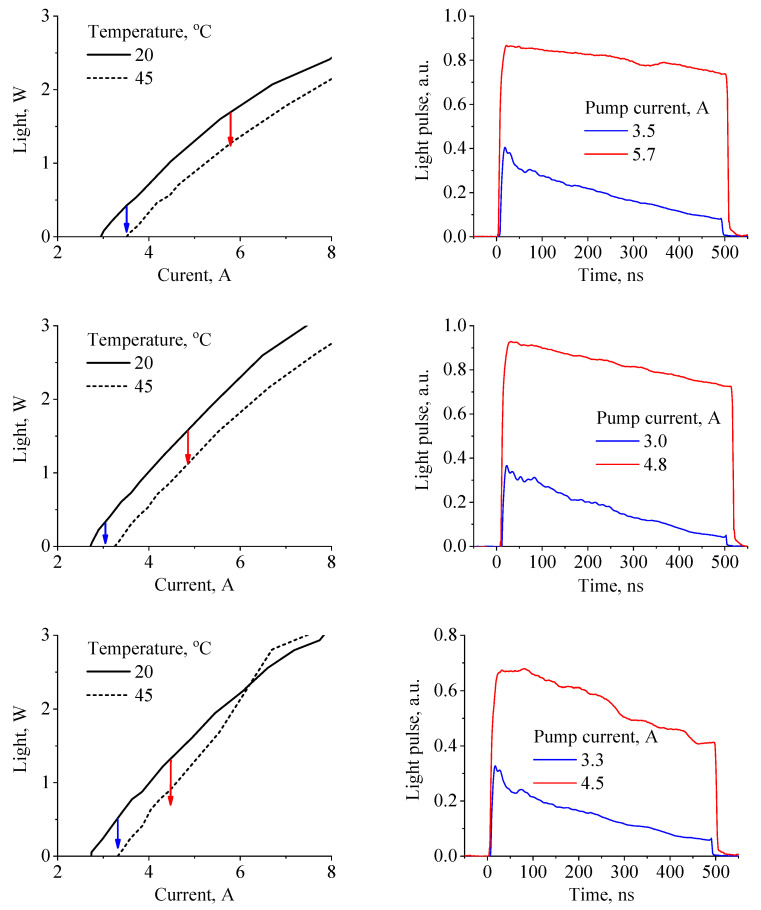
Left column—light–current characteristics for samples of InGaAs/InAlAs QCLs with different contact layers, as provided in [7]. Type C—upper row; Type B—middle and lower rows. Right column—time-dependent light pulses of the mentioned lasers under specified values of pump current. The degradation of the light intensity with time is also depicted in the left column using vertical arrows of corresponding color. One can see that the difference in QCL design does not play the same role for the intensity degradation.

## Data Availability

Data is contained within the article.

## Appendix A. Thermal Resistance of Heat Spreader Primitives

Consider the heat equation in the volume of planar and cylindrical heat spreaders (see Figure A1).

(A1) △T=0

The first model ((a) and (b) panels in Figure A1)) is a rod-like heat spreader with a height (*h*) significantly larger than its width (*w*). The nontrivial solution of the heat equation in a such geometry is a linear function:

(A2) $$T_{1D}\left(x\right)=kx+b$$

Using boundary conditions, one can obtain the temperature distribution in the rod-like heat spreader:

(A3) $$T_{1D}\left(x\right)=\frac{P}{\kappa Lw}\left(h-x\right)$$

The radial symmetry of the heat spreader leads to modification of the temperature distribution in the following form:

(A4) $$T_{rad}\left(r\right)=\frac{P}{\kappa L\pi }ln\left(\frac{R}{r}\right)$$
